# The Visual Effects of Intraocular Colored Filters

**DOI:** 10.6064/2012/424965

**Published:** 2012-08-21

**Authors:** Billy R. Hammond

**Affiliations:** Behavioral and Brain Sciences Program, UGA Vision Laboratory, University of Georgia, Athens, GA 30602, USA

## Abstract

Modern life is associated with a myriad of visual problems, most notably refractive conditions such as myopia. Human ingenuity has addressed such problems using strategies such as spectacle lenses or surgical correction. There are other visual problems, however, that have been present throughout our evolutionary history and are not as easily solved by simply correcting refractive error. These problems include issues like glare disability and discomfort arising from intraocular scatter, photostress with the associated transient loss in vision that arises from short intense light exposures, or the ability to see objects in the distance through a veil of atmospheric haze. One likely biological solution to these more long-standing problems has been the use of colored intraocular filters. Many species, especially diurnal, incorporate chromophores from numerous sources (e.g., often plant pigments called carotenoids) into ocular tissues to improve visual performance outdoors. This review summarizes information on the utility of such filters focusing on chromatic filtering by humans.

## 1. Introduction

It is often claimed that vision is improved when viewing the world through green windshields, red visors, rose-colored glasses, and so forth.Colored glasses and goggles are often used in (particularly outdoor) situations where precise vision is required. For example, amber goggles are used by sharpshooters to improve targeting, colored goggles are often used by snow skiers, and yellow glasses are marketed for driving and even promoted for improving driving performance at night. A number of new colored intraocular lenses have been created and are touted for their ability to protect against blue-light damage [1, 2], reduce glare, and enhance chromatic contrast [3, 4]. Despite widespread claims, sale, and use of colored lenses to improve vision, the empirical evidence of their efficacy is largely mixed. As noted by Provines et al. [5]:

*“The use of yellow filters to enhance visual performance has been proposed for more than 75 years. Many users, including some military aircrew members, are absolutely convinced that the yellow filters improve target acquisition performance; yet others are just as certain that they provide no improvement or even degrade performance.” *

Provines' et al., study [5] was designed to determine whether yellow lenses enhanced the ability to see approaching aircraft. The study had a null outcome, but the individual variability in results was large; the yellow filters decreased vision in some individuals but helped others. This outcome is characteristic of many studies on colored sunglasses; some studies find positive effects, some null, some negative (see the review by Clark, 1969 [6]). This paper focuses on explaining why past studies have reported such discrepant results and provides a simple answer; the spectral characteristics of filters matter and chromatic filters influence some aspects of vision but not others particularly those based on refraction. 

Laboratory and clinical tests of visual performance are most often based on basic assessments of acuity with stimuli that have not been carefully characterized. For example, Snellen acuity is determined largely by the axial length of the eye [7]. Filters might absorb the poorly focused light that blurs an image but such filtering is unlikely to improve the visibility of that image (filtering blur just makes less intense blur). If anything, it would simply reduce the luminance of the image and make it harder to see. In contrast, visual performance tasks that require seeing through a veil of scattered light (either within the eye, glare, or in the atmosphere, fog or haze) can be, potentially, improved by filtering.

Another limitation of past studies is that the filters themselves have not been well characterized. Often they are simply categorized by their color (a yellow filter was used). Of course, despite appearing similar in terms of their color appearance, filters can have dramatically different absorption characteristics. Past studies have not attempted to match the external filters that they are testing to the filtering characteristics of the filters that are actually within the eye itself (viz., the crystalline lens and macular pigment, MP). This general lack of attention to detail is unfortunate. Of course, we know an enormous amount about the characteristics of the human visual system. We also know an enormous amount about optics and filters. Hence, it is very easy to predict what aspects of vision will be improved by chromatic filters and what aspects would not (e.g., see the modeling by Bradley, 1992 [8]). 

The idea that chromatic filters can improve visual performance is based on a simple observation; intraocular colored filters are found widely in nature.

## 2. The Ubiquity of Colored Intraocular Filters in Nature

For over a century literally hundreds of papers have been published that describe the variety of colored filters that are found in the eyes of, largely diurnal, species. These filters are surprisingly homogeneous. Walls and Judd were the first to observe [9], for instance, that such filters invariably tend to be yellow (as opposed to retinal filters with other absorptive qualities, such as red filters). Many diurnal species share similar visual challenges such as veiling due to sunlight, seeing objects at a distance, and so forth [10]. 

Many species clearly use intraocular colored filters placed in various locations within the eye to solve these common visual problems [11–14]. For example, fish often have yellow corneas (pigmented corneas are almost exclusive to fish with the exception of some toads and the ground squirrel). Prairie dogs contain a yellow screening filter between their cornea and retina. Diurnal squirrels have intensely yellow lenses. The ellipsoids of cone inner segments of many species of birds contain colored oil droplets. Since the pigments are in the inner segments, light transduced by the photopigment in the outer segment must pass through, and be filtered by, these droplets. The pigments that color the oil droplets are primarily carotenoids such as zeaxanthin, astaxanthin, or galloxanthin (e.g., see the example of quail [15]). 

Walls and Judd [9] argued that this ubiquity of yellow filters (often based on the dietary pigments called carotenoids) was not accidental. Rather, that these filters were carefully matched to ecological niches that are often similar across species. 

One challenge of intraocular filters is that, unless a species is completely diurnal, light lost to the filtering can impede vision under low light conditions [16]. Puffer fish solve this problem by having “occlusable” corneas; the presence of the pigments depends upon the ambient light levels (nature's photochromic lens). In dim light levels, the pigment migrates to the periphery and in high light levels the pigment concentrates toward the center screening the distal retina [16]. This strategy is somewhat paralleled by the localized distribution of macular pigment (MP) in humans [17]. (Macular pigment is a yellow pigment that is found in the inner layers of the central retina. Derived from the diet, it is composed of carotenoids, specifically the xanthophylls lutein and zeaxanthin. Optical density of the pigments (screening mostly cones) can vary from as little as zero to over a log unit at peak absorbance (460 nm).) Humans have duplex vision, rods which operate in dim light and are mostly not screened by MP, and cones which operate at higher light levels and are screened by MP. 

## 3. Natural Selection and Vision in the Natural Environment

Until relatively recently, humans were either hunters and gatherers or agrarian spending most of their time outdoors. Life followed the rhythms set by the overall light cycle, diurnal and seasonally. Vision was based primarily on the need to see in the distance, items lit by natural sunlight, obscured by haze, and so forth. The mechanisms of how and what we see were therefore determined by how and what we saw for most of our species history: objects at a distance in the natural environment. 

Like many authors who study comparative evolutionary homology, Walls and Judd [9] argued that the ubiquity of intraocular yellow filters in nature (as opposed to retinal filters with other absorptive qualities, such as red filters) was evidence that *yellow* filters, in particular, play an important and immediate (i.e., confer a selective advantage) role in visual performance [10, 16, 18]. Douglas and Marshall noted [16] that since the intraocular filters of most vertebrates are short-wave (blue) absorbing, they probably also share similar functions. One obvious distinction is that species possessing intraocular yellow filters tend to be primarily diurnal as opposed to nocturnal where losing light due to filtration would simply be a disadvantage. Walls and Judd and later Nussbaum et al. listed [9, 19] four effects one could generally expect based simply on the optics of intraocular yellow filters.

1. To increase visual acuity by reducing the effects of chromatic aberration.
2. To promote comfort by the reduction of glare and dazzle.
3. The enhancement of detail by the absorption of “blue haze.”
4. The enhancement of contrast.

The first of these four functions outlined by Walls and Judd, commonly referred to as the acuity hypothesis, is widely stated in the literature as fact but has only recently been empirically tested [20, 21]. It is the only hypothesis of the four that suggests that yellow filters could actually correct *refractive* errors. Of the many optical hypotheses (there are others that will be expounded below) the idea that yellow filters influence refractive errors is the least tenable from an evolutionary point of view. In contrast, contrast enhancement, distance vision, glare reduction, these types of visual abilities would promote survival and successful competition. Empirical study, for example, has shown that the human yellow macular pigments are, in fact, related to these latter visual abilities [22–29]. In contrast, yellow intraocular filters do not appear to be strongly related to refractive error [20, 30]; filtering blur does not correct refraction. Hence, diet does not appear to overly influence modern visual problems like myopia. 

### 3.1. Visual Problems That Likely Result from Exposure to Modern Stressors

Refractive errors are certainly one of the more common visual issues that most individuals deal with [31]. In order to see objects in focus, light from the atmosphere refracts at two major anatomical points: the cornea (first refractive surface) and the crystalline lens (second refractive surface). Many optical surfaces, such as the human cornea, are unmoving and provide fixed focal lengths that vary from each other depending on whether light is passing through the corneal center or a more peripheral location. The human crystalline lens, however, is more flexible and can change its shape, which consequently changes its focal length. The end result of having these two optical structures, one fixed and the other accommodative, is that humans can maintain a variety of objects in sharp focus, despite the fact that these objects may be at different distances from the eye. 

Refractive errors can occur for a number of reasons, but the most common causes can be described as occurring in three, nonmutually exclusive basic varieties: those arising from the cornea, those arising from the crystalline lens, and those that arise from aberrations in the shape or length of the eye. For example, astigmatism is a common refractive error that is caused by an uneven corneal surface. If the corneal surface is uneven, refractive power is uneven across different meridians of the corneal surface, and fine visual detail is often lost. Although astigmatism is relatively common, its incidence tends to increase with age [32]. Presbyopia is an age-related condition that occurs when the crystalline lens loses its ability to change shape and accommodate objects at near to the viewer. Myopia (nearsightedness) and hyperopia (farsightedness) are common refractive errors that most often results from having an eye with an axial length that is too long (myopia) or too short (hyperopia). In the myopic eye, increased length of the eye results in an increased distance between the lens and the neural retina. Consequently, an object that should fall into sharp focus on the retina itself will fall into sharp focus in front of the retina. Given that light spreads after the focal point, the light that falls on the retina in a myopic eye has lost focus by the time it can actually be transduced. Individuals with myopia are thus termed “nearsighted,” which means that objects must be moved closer to viewer to be viewed in sharp focus. In the case of the hyperopic eye, the decreased length of the eye results in a decreased distance between the lens and the neural retina. Consequently, an object that should fall into sharp focus on the retina would, if the tissue were transparent, fall into sharp focus behind the retina. Consequently, the light that hits the retina is not yet perfectly focused and the resulting image is blurred. 

A high proportion of the population (approximately 153 million people) have uncorrected refractive issues [33]. The number of individuals with corrected refractive issues is difficult to ascertain but is estimated in the billions. Given the fact that vision evolved outdoors, it is undoubtedly the case that refractive errors were, historically, rare, given the fact that presence of a severe refractive error would make sight, especially at a distance, difficult. 

Lack of acute vision would certainly influence the chances that an individual with a refractive problem would survive until reproductive age. Refractive errors are, in this way, like many facets of modern life that are inconsistent with our physiology. For example, it is likely that the liver produces cholesterol because cholesterol is an essential component of cellular membranes, and fat sources were likely often rare before the agricultural revolution. Modern diets contain a surfeit of saturated animal fat which has been linked to increases risk of cardiovascular disease and obesity, even in children. Similarly, most visual tasks outdoors (say hunting or farming) do not require close scrutiny of near objects. Indoor tasks like reading, however, require controlled exertion of extraocular muscles in order to follow small lines of script for long periods. This action, over years, can result in increased axial length (hence, the high incidence of myopia in professions that require extensive reading; [34]). So much near work in a visual system that evolved to mediate vision at a distance has created numerous problems. Myopia, for instance, is pandemic. In Singapore, 20% of children are myopic with the prevalence exceeding 70% by the completion of college [35]. In the United States, the prevalence of myopia has increased from about 25% in the early 1970s to about 43% in the early 2000s [36].

The shift from a rural- to an urban-based economy in the last 100 years is widely thought to have accelerated increases in the prevalence of myopia [37]. There is a general consensus that the etiology of myopia includes genetic predispositions (e.g., variations in the toughness of the scleral connective tissue make axial length more or less modifiable) combined with environmental stressors (such as a lack of feedback from visual signals that help regulate ocular growth [38]). It seems clear, however, that the genetic component itself (whatever size) can be swamped by environmental factors, which vary from population to population. Young et al., [39, 40] originally showed that Alaskan Eskimos had extremely low incidence of refractive errors until first exposed to a compulsory education and acculturated to a Westernized style of life. Similar observations have been made with Australian Aborigines [41]. Heritability estimates on these populations, when based on parent-offspring calculations, are very low (e.g., *h* ^2^ = 0.10). In contrast, heritability estimates based on sibling concordance are very high (*h* ^2^ = 0.98). As noted by Guggenheim et al. [42], this suggests that “environmental factors (dominate) any influence of genetics in determining refractive error.” Such a conclusion is warranted but “environmental factors” historically did not include the visual activities that now dominate our days: reading, computer use, and so forth. Ironically, a return to a more natural lifestyle may be able to attenuate many visual problems associated with more modern visual tasks. Dirani et al., have shown that increasing outdoor activity significantly reduces myopia incidence in teenagers from Singapore and this risk reduction was independent of near work [43]. 

Taken together, it is probably safe to conclude that refractive error is a modern visual problem and a problem that natural intraocular filters did not evolve to correct. 

### 3.2. The Concentration of Intraocular Colored Filters, Namely Macular Pigment, Is Too Low to Solve Visual Problems Most Individuals Encounter Outdoors

 For an expanded recent discussion of macular pigment see the review by Sabour-Pickett et al., and Kijlstra et al., [44, 45]. For the purpose of our discussion here, however, several points are worth noting. There is about 12 mg of lutein (L) and zeaxanthin (Z) in about one cup of green leafy vegetables (like spinach) [46]. The average intake of LZ in the American population is 1-2 mg per day (2 mg is about the 80th percentile) [47]. Hence, one cup of spinach a week is equivalent to the average amount of green leafy vegetables most Americans consume. Compare this to the average intake of LZ for groups that are largely agrarian or hunters and gatherers; Le Marchand et al., for instance, noted that the average intake of LZ for Fiji Islanders was about 20 mg/day (about 10X the American norm) [48]. It is not surprising that the average American diet is deficient in carotenoid-rich foods. What is perhaps surprising is that it is so dramatically deficient. For instance, the optical density of macular pigment (measured at peak absorbance, 460 nm), in an individual with a very good diet, has been measured to be as high as an optical density (OD) of 1.6 [17].

For most of the population, however, the average levels of MP density are quite low. For example, Hammond et al., measured MP density in a large (*n* = 660) sample of young subjects [49]. The average MP optical density (OD) in that sample was about 0.24. It is likely that the overall poor dietary habits of the American public are likely to simply get worse. If the eye, like most of human biology, depends on optimal dietary intake for optimal function, then it is likely that many are not seeing nearly as well as they could.

### 3.3. Characterizing Visual Function: Beyond Refractive Error

Good acuity and refractive state predict many aspects of visual performance. Unfortunately, however, there are many aspects of vision that are not well predicted by refractive state. Some examples include photopic and scotopic sensitivity, color perception, depth perception, object perception, hyperacuity, chromatic contrast, temporal vision, visual motor skills, glare discomfort and disability. As an example, glare disability is caused by exposure to a bright light that is in excess of an individual's adaptive state (e.g., you are more sensitive to light when dark adapted). Such light will scatter within the eye causing general degradation of visual function. 

Intraocular scatter is not related to refractive state. This was demonstrated in a large sample (*n* = 2422) of European drivers studied by Van Den Berg, et al; young subjects with very good acuity can have high levels of degrading intraocular scatter [50]. Glare due to sunlight is a common source of accidents during the day [51]. The disability and discomfort that arises from exposure to sources like the sun and headlights is caused by light scattering within the media of the eye. This scattered light causes a veil that obscures vision and will temporarily blind a driver. Visual problems due to glare increase significantly as we age and are a common reason that older individuals refrain from driving at night. This precaution is well founded; the statistics on night-time accidents show that most are, in fact, caused by glare arising from bright headlights either in the front or rear of the driver. For this reason, it is often suggested [52] that glare disability testing be added to the requirements for a driver's license, especially for older drivers.

Visual problems due to glare have been a problem that has affected human vision for as long as there have been humans. Seeing in the distance, for example, is limited by many of the same factors as those that produce glare (e.g., scattered sun light). Hence, evolution has provided a remedy for reducing intraocular scatter, intraocular filters. Filtering by macular pigment, for instance, cleans up an image by absorbing the scattered light that does not contribute usefully to visual processing. There is a large body of empirical scientific evidence showing that supplementing these pigments will reduce the disability and discomfort caused by intense light [22–25]. 

High intraocular scatter does not only cause problems at high light levels. In fact, intraocular scatter reduces vision across a number of dimensions. For example, chromatic discrimination can be reduced due to bright light desaturating colors [28]. Color enhances the coding of images at the input stage by facilitating the detection of borders. Isoluminant edges (i.e., edges defined only by chromatic differences) are common in natural scenes and when viewing objects at a distance since the distance itself tends to equalize differences in luminance that would otherwise have demarcated an edge if the object was closer [53].

Of course, a large variety of factors can exacerbate intraocular scatter [50, 54–57]. Age and ocular disease are strongly associated with worsening scatter. Many neurological conditions (e.g., mild traumatic brain injury as discussed later) are associated with increased sensitivity to light (e.g., migraine sufferers) and glare disability. Indeed, some ocular conditions (like vitreous turbidity, corneal dystrophies, etc.) are largely defined by intraocular scatter. Intraocular implants that are used to replace cataractous lenses are often associated with increased glare problems [58]. An important factor here appears to be how well the operating physician clears the cataractous natural lens (scatter arises from the rough junction of the implant and the remaining capsule). Indeed, even laser corrections (like LASIK) for myopia can significantly increase intraocular scatter [59]. 

Light does not obviously just scatter within the eye itself but also within the atmosphere [60–62]. This scatter is inversely proportional to wavelength (higher energy light at shorter-wavelengths scatters more than low-energy light at higher wavelengths) as described by Rayleigh's famous equation (wavelength raised to the negative fourth power, [63]). Good vision outdoors depends on the light source (typically the sun when outdoors), how objects reflect light, and actual interference by light (the composition of air light). Blue haze [26] is an example of this last phenomenon and is expounded more later in the section on visibility.

Both the sun and haze are strongly “blue.” Sky light obviously looks blue. Haze will occasionally look blue (such as the Blue Mountains, or purple mountains majesty). Even when it does not appear a blue hue, however, it is clearly still short-wave dominant (e.g., the earth appears very blue from space). This has been shown by careful atmospheric measures of haze (reviewed by Wooten and Hammond [26]). It is this haze that will limit visual range. 

One major function of either internal intraocular filters (like macular pigment) or external colored contacts is to improve visual range by absorbing the haze projected with an image that is focused on the retina. By absorbing out the haze portion of the image, the resultant view is “cleaned up.” 

## 4. The Visible Spectrum and the Photopic Sensitivity Function

 So what is the downside of colored filters and vision? The major downside is simply that they reduce the amount of usable light to the eye. Light is, after all, the only stimulus for vision (obviously no vision is possible in total darkness). Hence, reducing stimulus input, especially under low light conditions, can simply be detrimental. For example, many nocturnal species possess an intraocular retroreflector (termed the tapetum lucidum) that reflects visible light forward in order to maximize light capture at night (the reason a cat's eyes “glow” at night). Such reflection gives photoreceptors a second opportunity to respond to light photons but also greatly increases problems due to intraocular scatter. Those costs, however, are outweighed by the greater benefit of seeing at all when light levels are very low. 

How is light loss by intraocular filters solved in nature? One strategy is simply niche specificity. Many diurnal animals dramatically limit their visual activity at dawn/dusk and night. For example, if you are a bird with mostly cone photoreceptors and colored oil droplets, you simply hide/sleep at night and hope a nocturnal predator does not find you. Nocturnal mammals, like rodents, have retinas that are rod dominant. Humans, occupying a diversity of environmental niches, solve the light loss problem by simply concentrating their intraocular chromophores in the area of the retina mostly used during the day; macular pigment accumulates in front of the cones and (largely) not in front of their rods. The slow oxidation of crystalline proteins in the lens, however, also slowly turns the lens yellow. Since the lens screens the entire retina, another strategy had to be employed. This strategy was likely a differential sensitivity to the visible spectrum; humans are relatively insensitive to light that is filtered by their own intraocular filters.

**Figure 1:**
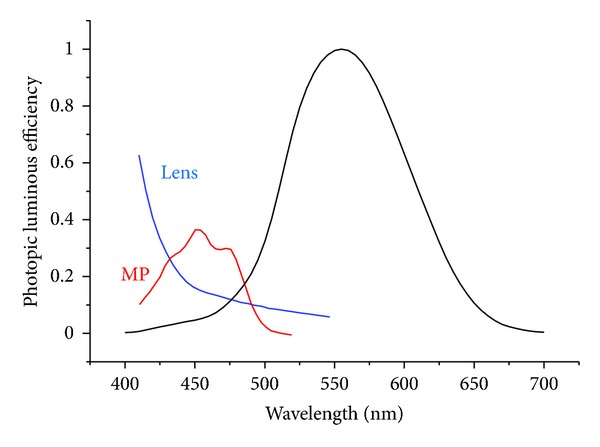
The photopic (light-adapted) spectral sensitivity function plotted next to the internal colored filters, the yellow crystalline lens, and macular pigment (from Wyszecki and Stiles, [64]). Note that the colored filters do not significantly overlap the photopic spectral sensitivity function.

**Figure 2:**
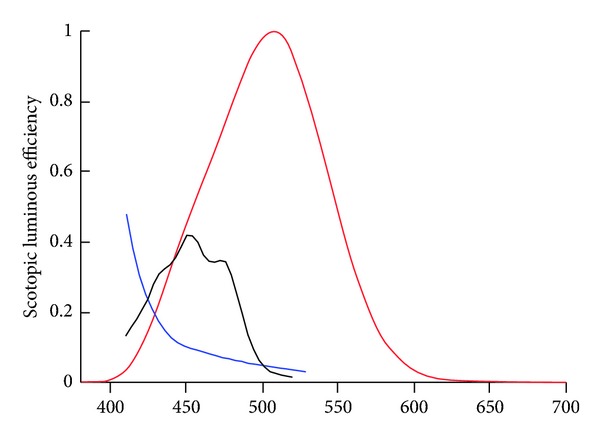
The scotopic sensitivity curve plotted with MP and lens absorbance (Wyszecki and Stiles, [64]). This curve is significantly shifted to the short-wave end as compared to the photopic curve above. Note that the lens is still not a major filtering impediment to dark-adapted sensitivity (fortuitous since it screens rods). MP density, however, does significantly overlap with the curve. MP is localized within the retina as to mostly screen the cones and not the rods to obviate this problem.

Humans, like most animals, are sensitive to a very limited portion of the overall electromagnetic spectrum. Although not exactly the same for every individual, the general range that comprises visible light ranges in wavelength from about 400 to 700 nm. We are not, however, equally sensitive to this entire range.  Figure 1 shows the photopic (light-adapted) spectral sensitivity function plotted next to the internal colored filters, the yellow crystalline lens, and macular pigment (from Wyszecki and Stiles, 1982 [64]). Note that the colored filters do not significantly overlap the photopic spectral sensitivity function. This is less true for the scotopic visual function as shown in Figure 2.

### 4.1. Cone Mechanisms Mediating the Photopic Spectral Sensitivity Function

As a general descriptor, vision operates by breaking an image down into its component parts and then different sections of the brain reconstitute those sections into a meaningful gestalt. Some general observations about this process are worth noting (for a general review see Schwartz, 2010 [65]). The first, and somewhat most obvious, is that most of what individuals think of as vision occurs in the brain. The eye is largely a detector that turns light waves into neural signals. The brain turns those signals into meaningful percepts. For example, as Newton originally observed, 670 nm light is not red per se. That light wave is simply transduced by long-wave cones and sent as a signal to the brain (carried as a signal along the red-green opponent color channel). The brain (e.g., the extrastriate area V4) turns that wavelength into the perception of red. The reconstituting of visual information is a major feat of the brain and commands considerable neural real estate (more than any other sensory modality). 

Actually, as optics go, the eye is not optimal. For example, it is often moving in rapid jumps (called saccades) and is obstructed by internal debris (e.g., vitreous floaters), has light obstructing layers (inner retinal layers and vessels anterior to the outer segments), relatively large blind spots (optic nerve), and so forth. What the eye does accomplish, however, is the differential processing of light. For example, the differential sensitivity of the cones segregate the signal by wavelength and this allows the inchoate encoding of color. The cones process the higher spatial frequencies, wavelength information, detailed spatial information, and so forth and send this information to the brain down a specialized channel called the parvocellular pathway. (To be more specific, inputs from the L and M cones are processed antagonistically by the midget ganglion cells. These cones, together with ganglion cells, form the parvocellular pathway. A different parallel system, the koniocellular system, is activated by the S cones and is responsible for the opponent yellow-blue channel. L and M cone inputs, processed additively by the parasol cells, also contribute to the largely rod-driven magnocellular pathway, which mediates achromatic spatial and temporal functions. The parvocellular pathway also responds to variations in luminance at high spatial frequencies and slow temporal stimuli (up to 1 Hz).) The rods process low spatial frequencies, motion, and so forth and this information is sent down a separate channel called the magnocellular pathway. 

One reason for such complexity is to allow humans to see well under a huge variety of circumstances. Spatial vision, for instance, is mostly processed by cones. Light that is most often used, however, for demarcating objects is in the middle of the visible spectrum and is processed by the mid-and-long wave cones. The short-wave end of the spectrum is useful for color processing but scatters too much in the atmosphere to be useful for spatial analysis. Hence, short-wave (blue) cones contribute mostly to better color vision (the chromatic channel) but are too sparse to contribute usefully to the overall photopic spectral sensitivity curve. In addition, it is mostly the M- and-L cones that contribute to the luminance channel. This is the channel that mediates spatial vision. 

### 4.2. Effects of Natural Intraocular Filters on the Chromatic and Luminance Channels

 Yet another description of how visual light signals are parsed by the visual system is the chromatic and luminance channels. The luminance channel simply adds the signals from L and M cones [64–66]. Under most conditions, the S cones do not contribute to luminance [67, 68]. (The most common method for measuring macular pigment is heterochromatic flicker photometry. This method utilizes M and L cones and the luminance pathway. This is why the method is valid. The chromatic channel compensates for MP density (see the following section) and, if it contributed to the technique, would confound objective measurement of the pigments.)

 Any filtering of the luminance channel will simply lead to decreased visual function. The visual system, however, can compensate for light loss within the chromatic channels. For example, the S cone system increases gain to offset filtering by the yellowing crystalline lens and macular pigment [69]. 

### 4.3. The Visual System Can Correct for Light Loss due to Internal Colored Filters by Ramping up Sensitivity: Compensation 

Sensitivity regulation is probably one of the more fundamental characteristics of the visual system. For example, despite going from about 90 million rod photoreceptors when one is around 20 years of age to about 60 million when one is about 60 years of age, there is relatively very little loss (1-2%) in scotopic (rod-mediated) sensitivity [70]. On a daily basis, the visual system must regulate overall sensitivity in order to deal with large variations in ambient lighting. Such regulation, partly due to pupillary diameter, but more significantly due to receptoral and/or postreceptoral gain mechanisms [69], is relatively, spectrally, nonspecific. Spectrally specific regulation, however, defined by specific mechanisms, is also often necessary. von Kries originally described [71] sensitivity regulation of cone mechanisms for the purpose of maintaining color constancy. More recently [72], Neitz et al. showed that wearing colored filters could shift unique yellow by several nanometers. This shift persisted for 1-2 weeks after discontinuing use of the filters. Correcting for screening by colored goggles is specific to wavelength but not location (the entire retina is screened). Spatially discrete compensation has also, however, been described. For example, Sunness et al. showed that retinal sensitivity in patients with early AMD was constant when comparing sensitivity over drusen and nondrusen areas [73]. Compensation has been studied using behavioral responses (e.g., psychophysical measures of sensitivity; Stringham et al. [74, 75]). These responses, however, are mediated by specific underlying, and relatively independent, visual mechanisms that can often be determined through psychophysical means [76, 77]. For example, Hibino originally showed [78] that compensation for MP influenced the Y-B opponent system without influencing the R-G system despite the fact that MP absorbance clearly influenced the G lobe of the R-G system. 

According to the principle of univariance, receptors only respond to the light they receive and cannot differentiate whether such light is attenuated by the lens or MP or a colored contact lens (the key here is that all three are stable, unlike glasses which are removed regularly during the day and would defeat gain adjustments). It is for this reason that compensation mechanisms for each might be very similar (i.e., relegated to the Y-B opponent system [75]). The mechanisms for how the visual system compensates for lens and MP has been studied.

#### 4.3.1. Macular Pigment

Snodderly et al. originally showed [79, 80] that the distribution of L and Z within the central retina was highly specific. Using two-wavelength microdensitometry in monkey retinas, Snodderly et al. found that MP was concentrated in the Henle fiber layer, peaked in the center of the fovea, and decreased rapidly and monotonically to a low constant that did not absorb visible light at approximately 1 mm (3° visual angle). This basic pattern has been confirmed in other ex vivo studies on monkeys and humans. For example, Hammond et al., using heterochromatic flicker photometry (HFP) with small test stimuli, measured MP spatial profiles on 32 subjects [17]. Their data, as well as other recent HFP data [81], showed that MP declined as an exponential function with eccentricity and was symmetric in the vertical and horizontal meridians of the retina. In addition to the highly specific spatial distribution of L and Z within the eye, the spectral absorption of the pigments is also distinct. MP absorbs light from about 400–500 nm reaching maximum peak absorption at around 460 nm [79]. There also appears to be wide individual differences in both the spatial distribution and peak optical density of MP [17, 81]. For instance, some subjects appear to have very low levels of MP, whereas others have MP in such high quantity that most of the short-wave portion of the visible spectrum is effectively screened from the photoreceptors [17]. This population distribution of MP is similar to that seen when examining individual differences in serum levels of L and Z and dietary intake of L and Z [82, 83]. As with dietary patterns, which are relatively stable [84], individual differences in MP optical density (OD) persist over time. Hammond et al., measured the MP OD of ten subjects over periods ranging from 1–16 years [17]. They found that the MP of these subjects changed very little suggesting that, in the absence of significant dietary change, individual differences in MP OD are stable. It also widely concluded that average MP levels do not change with age [85].

#### 4.3.2. Compensation for Screening by the Macular Pigments

 At the center, MP values are often over 1.0 optical density (OD) relative to a parafoveal reference with occasional individual subjects having peak densities at the foveal center estimated to be as high as about 1.6 OD units at 460 nm [17, 81]. Although such dense pigmentation serves some positive functions [44, 45], it raises interesting perceptual issues. The uneven distribution of MP across the retina produces large variations in the distribution of light (between about 420 to 520 nm) incident on the central photoreceptors. For example, for individuals with high densities of MP, the transmission of 460 nm light incident on the photoreceptors can vary by as much as 97% within a few degrees eccentricity. Based purely on optical filtering, such individuals should perceive significant shadowing in their central visual field, which does not usually occur. The visual system must somehow compensate for this dramatic and variable filtering by the MP.

Using a hue-cancellation method, Hibino originally showed (*n* = 2) that the sensitivity of the blue component (relative to the yellow) of the Y-B opponent system (but not the R-G system) was essentially constant across the retina despite variation in MP density. Hibino concluded that the visual system must increase the gain of the B component in order to offset differential filtering by MP across the retina ([78], also see [75]). 

#### 4.3.3. The Crystalline Lens

The lens is the most transparent tissue within the body. Most cells, even those that are avascular (e.g., bone cells), are opaque due to their organelles, other absorbing chromophores, and high optical scatter arising from an uneven distribution of refractive elements. In contrast, the young lens has nearly no light-absorbing pigments, and the packing of the 1000 layers of clear crystallin cells is highly ordered. Controlled apoptosis during development removes optically dense organelles, leaving the cell essentially alive but without the ability to regenerate or repair damage. As such, any damage to cells within the lens (usually due to oxidative modification of crystallins) simply accumulates with age causing the lens to slowly opacify. This opacification is both distinctive and relatively stable across the lifespan. It is well known, for instance, that lens absorbance is strongly, and inversely, related to wavelength [64, 86]. Another important feature of lens OD is that absorption increases linearly with age and that individual variation is large and tends to be relatively uniform across the life span [87]. For example, Hammond et al., measuring lens OD at 440 nm in a young sample, found a range of lens OD from 0.06 to 0.99 [88]. Most studies find that lens density ranges by a factor of, at least, 2-3 when measuring even young subjects [89, 90]. 

#### 4.3.4. Compensation for Screening by the Lens

One of the primary determinants of color appearance is the dominant wavelength reflected from a given object. As such, the dramatic increase in absorbance of short-wave light over the lifespan might be expected to influence the perception of the color blue. Nonetheless, there seems to be strong evidence that for most individuals color perception is relatively constant across the lifespan [69]. For example, Hardy et al. showed that color naming did not change according to lens OD [77]. Under normal circumstances, age-related compensation for the lens probably occurs slowly as lens density increases (by about 0.01 OD per year). Delahunt et al., however, studied this process under the unique circumstance of where lens OD changes quickly, cataract removal [91]. These authors showed that removal of a cataract causes large changes in color perception but, after a few months, color constancy is restored.

#### 4.3.5. General Compensatory Mechanisms

There are obviously a number of potential mechanisms by which the visual system could compensate for spatially and/or spectrally discrete filtering. For instance, neural algorithms could exist that would simply accept the altered input and construct a visual field of equal brightness. For instance, there are lower-level inhomogeneities that are clearly compensated for at higher levels. For example, filling-in phenomena (e.g., to correct for scotomas [92]) have been described [93] as an active cortical process of providing information (based on surrounding cues) to fill in a discrete area where information is lacking or deficient. Compensation related to color appearance has also been described as being mediated by cortical mechanisms [72, 91]. In contrast, *sensitivity regulation* is generally assumed to be mediated at the retinal level (i.e., essentially a multiplicative process that independently regulates sensitivity of the three cone mechanisms generally according to Weber's law). At this level, compensation appears to be directly linked to incident light. Thus, any change in illumination (due to filtering, ambient light levels, etc.) causes relatively rapid compensation in the outer retina [94]. One could predict that compensation at the retinal level would therefore correct for all filtering (i.e., it would respond in a manner similar to changes in ambient illumination). If MP does not influence the R-G channel (as shown by Hibino, 1992 [78]), it therefore seems unlikely that compensation for MP is mediated by simple sensitivity regulation. Rather, compensation for MP appears to be mediated by, at least, one of the major parallel pathways, the Y-B channel. These channels, of course, reflect retinal, postreceptoral, and cortical processing. There is some evidence for the idea that compensation for MP and lens filtering is relegated to the Y-B, as opposed to the R-G, channel [75, 78]. (1) The Y-B system shows complete compensation for MP but the R-G system shows zero compensation [78]. (2) The *π*-1 mechanism shows complete compensation across the retina in young subjects (this is probably just the B component of the Y-B system using a different method) [74]. (3) Werner and Schefrin show [95] some compensation based on the locus of the achromatic (“white”) point across a large age range. Lens density was almost certainly increasing across age (although this was not measured). (4) Similar to Werner and Schefrin [95], Delahunt et al., showed [91] partial compensation for constancy of the white percept before and after cataract removal.

## 5. Visual Functions Influenced by Intrinsic and Extrinsic Colored Filters

### 5.1. Luminance

 As noted, filtering will decrease input to the M and L cones, which input to the luminance channel, and will negatively influence spatial vision under low-light conditions. This is likely the basis for why intraocular filters restrict filtering to the short-wave region of the spectrum (or why the “best” design of extrinsic filters is yellow, see Figures 1 and 2). The fact that luminance is related to spatial determinations like recognition acuity is well known.

### 5.2. Intraocular Scatter and Vision

Although scattering within the eye is most often associated with glare issues, scattering is a linear phenomenon that degrades vision under even low light (it is just more obvious in high-light conditions). This is easily seen when viewing the spread of light in a normal eye when viewing a point source (the point spread function). The issue here is that light must pass through the cornea and lens which do not perfectly pass such light (this fidelity of passage is often represented when testing external lenses by the modulation transfer function). This degradation (due to scattering and various aberrations) is often represented (when viewing a small spot of light; an extended source is described by a line spread function) by the point spread function. The point here (to risk a pun) is that any degradation reduces visual function. It is certainly not clear that colored filters would reduce the low-level aberrations and scatter that can degrade very detailed vision. It is important, however, to realize that scattered light represents a continuum: scatter increases linearly with intensity. Discomfort is not as linear and is obviously highly linked to the adaptive state of the subject (e.g., the discomfort from the light of the refrigerator in middle of the night). Disability is likely to be more linear and more linked to simple scatter. 

In any event, scattering (and generally degradation of the visual signal) degrades vision at all levels of intensity. Its most deleterious manifestations, however, are most obvious at high light levels. Scatter within the eye is of optical origin; the cornea and lens account for the majority of intraocular scatter. As discussed, this scatter has a general degrading effect upon vision. The most obvious examples of deleterious effects of intraocular scatter are glare disability and discomfort. 

### 5.3. Glare Discomfort

 Glare discomfort was studied by Stringham et al. [24, 25] and Wenzel et al. [96]. A major complaint for many AMD patients is visual discomfort as a result of exposure to even moderate lighting [97]. This is termed “photophobia”, or “discomfort glare”, and refers to discomfort, or, in extreme cases, pain on exposure to sufficiently intense light. Stringham et al. showed [24, 25] that thresholds for photophobia responses (squinting of the eyes in reaction to an intense light) were much lower for lights of short wavelengths (those in the blue region of the visible spectrum), compared to lights of middle (green) or long (red) wavelengths. In other words, it took much less light energy to elicit an aversive response when the light was of a short wavelength. Interestingly, the action spectrum for photophobia (after correction for MP and ocular media absorption) was shown to approximate both the threshold retinal damage function for rhesus monkeys determined by Ham et al. [98, 99] and the action spectrum for aerobic photoreactivity of lipofuscin (thought to act as a photosensitizer for the generation of reactive oxygen species in the retina [100]). It appears, therefore, that photophobia is a behavioral mechanism that is biased to protect biological tissue from potentially damaging short-wavelength light. With regard to MP level, subjects with higher levels of MP were shown to tolerate more short-wavelength light energy before the photophobia threshold was reached. A similar result was found in another study of photophobia in which thresholds to a broadband white light (containing much short-wavelength energy) versus an orange light (containing no short-wavelength energy) were compared [24]. Overall, the subjects were shown to be more sensitive to the broadband white light, but those subjects with higher levels of MP were able to tolerate higher levels of that light when viewed centrally (filtered by the MP) compared to peripherally. Conversely, for the orange light, the subjects were shown to be very similar in their photophobia sensitivity for central versus peripheral viewing conditions. From a functionality standpoint, these studies indicate that MP increases the bandwidth of comfortable visual operation via its action as a passive filter. For subjects with relatively high MP levels, a conservative estimate of this effect is roughly 0.5 log units (over three times the amount of broadband light energy tolerated) compared to those with very little or no MP. Wenzel et al. supplemented four subjects with lutein esters (Xangold, 60 mg) for 12 weeks and found that increases in MP density led to proportional improvements in photophobia [96].

### 5.4. Photostress Recovery

Photostress can be thought of as an after effect of extreme glare disability. After being exposed to a bright light, it takes time for the visual system to readjust sensitivity to the new conditions. This phenomenon occurs, in part, because the light-sensitive photopigments used for vision are bleached by light (analogous to exposing camera film) and require time to return to their previous configuration. While this recycling procedure occurs constantly in the visual system, the sudden deficit of intact photopigments following a photostressor causes temporary blindness. By filtering the energetic short-wave portion of this bleaching light, any intrinsic or extrinsic filter can reduce the amount of light actually reaching (and consequently breaking up) the photopigments and decrease the amount of time required to recover from a photostress event. Photostress is not only mediated by photopigment isomerization. Sudden exposure to any bright light that exceeds a subject's adaptive state can lead to temporary loss of vision.

A sudden loss of vision can be quite debilitating under certain circumstances. One obvious example is when driving; increasing photostress recovery speed by just 5 seconds (as was done in young subjects in Stringham and Hammond [23] by increasing MP density by 0.16 OD units from supplementation) can translate to about 440 feet when traveling 60 mph. Any filter that reduces a photostressor would speed recovery. This was shown by Hammond et al., 2009 and 2010 studying the visual effects of implanting yellow intraocular lenses [3, 4].

### 5.5. Glare Disability

In glare conditions, this forward scattering of light can be very conspicuous and results in the reduction of an image's contrast, thereby reducing visibility. This is a common visual deficit experienced in situations such as night driving due to exposure to bright headlights. The elderly are especially vulnerable to impaired vision in these situations, as structural changes in the crystalline lens lead to greater light scatter. Filters, either intrinsic or extrinsic could, in theory, help absorb scattered light, thereby improving visibility in glare for 3 reasons. (1) The foveal cones are screened. (2) The absorption spectrum of our optimal contact would cover roughly one-third of the visible spectrum (like MP and the lens), so is capable of absorbing a visually meaningful amount of scattered light. (3) The “kind” of light that would be targeted (short wavelengths) is relatively less important to the visual system in terms of luminance, or brightness, than middle or long wavelength light. In most cases, therefore, a tinted lens would not negatively impact the visual detection of a target. 

Stringham and Hammond [22] investigated the role of MP in improving visibility, as opposed to simply reducing discomfort, in the presence of a glare source. Thirty-six subjects with a wide range of MP values (from 0.08 to 1.04 log optical density) participated in their study. Visual performance was assessed as the ability to detect a 100% contrast grating stimulus (a black and white striped pattern) under intense glare conditions. The glare stimulus was an annulus (concentric with the target stimulus) that consisted of either broadband (i.e., “white”) light or monochromatic light ranging from 460 to 620 nm. The subjects' task was to increase the glare intensity of the annulus to the point when the grating target just disappeared. As expected, for subjects with high levels of MP, the scatter effect was greatly reduced for the short wavelength monochromatic lights. Interestingly, for the broadband white light, an even stronger effect of scatter reduction was found. Subjects with higher MP were able to withstand much more of the white light glare before losing sight of the target (*P* < 0.001). This finding suggests that the filtering effect of MP integrates across wavelengths, and thus MP is apparently very effective at relieving disability glare under broadband illumination. The authors suggested that a filtering mechanism, specific to MP's absorption spectrum, is responsible for the relation between MP and disability glare in such conditions, as no relation was found between MP and glare sources composed of wavelengths outside the absorption spectrum of MP (e.g., 620 nm). In an attempt to extend these cross-sectional findings and determine a possible causal relationship between MP and disability glare, Stringham and Hammond [23] measured changes in MP and disability glare following a 6 mo daily supplementation regimen of 10 mg of lutein and 2 mg of zeaxanthin. In a linear fashion, subjects' MP levels increased during the supplementation trial (average increase of 0.16 log optical density after 6 months of supplementation), and a reduction in disability glare commensurate with MP increases was also found. These results confirmed a causal relation between MP and disability glare (and have been recently replicated in [101]). In fact, improved visual performance corresponded to subjects' ability to withstand an average of 58% greater intensity of the glare source before losing sight of the target.

### 5.6. Chromatic Contrast Enhancement

**Figure 3:**
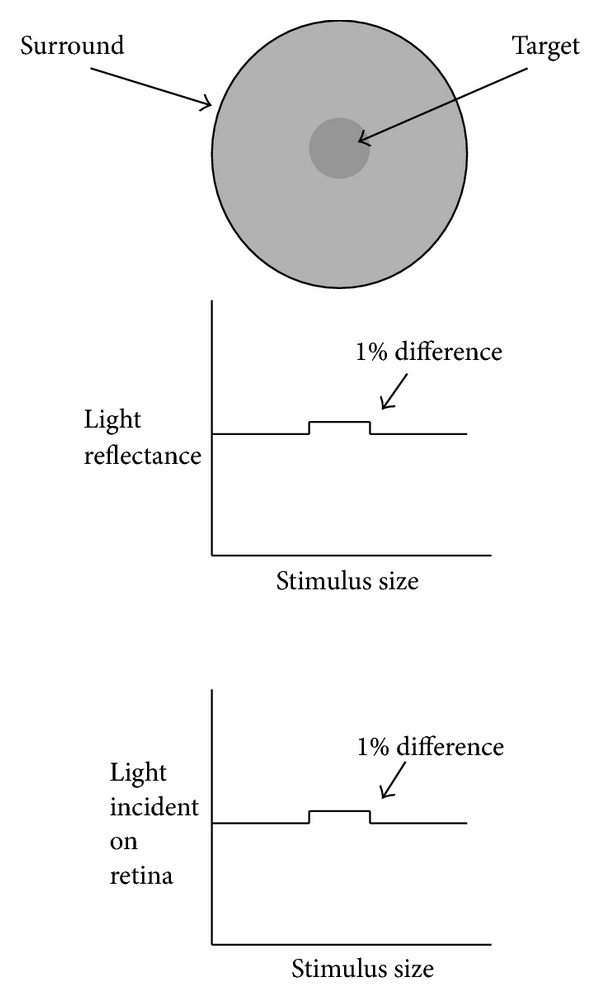
The example above shows an achromatic test target and surround of nearly the same luminance. The central target becomes visible when either the target is just slightly brighter or the surround is just slightly darker. This very small change in brightness is enough to create a luminance edge (a phenomenon called brightness induction). These experiments are typically done with achromatic stimuli like those shown above but a similar effect would hold for colored stimuli.

Walls and Judd [9] also argued that colored intraocular filters enhance chromatic contrast. Enhancing contrast is a very important aspect of spatial vision, particularly as they apply to edges. Edges drive vision (the retina has been described as a “contrast engine”) and the visual system is organized to accentuate edges (e.g., lateral inhibition in receptive fields). The diagram shown in Figure 3 provides an example.

As noted, edges have an exaggerated importance in many perceptual tasks. Edges define the boundaries of objects and are therefore necessary to segment, register, and ultimately identify objects in a scene. Retinex algorithms (a major theory of color vision), for example, emphasize the importance of color borders. Simple cells within the cortex are maximally sensitive to edges of a given orientation and lateral inhibition within the retina accentuates discontinuities within our visual field. Anything that accentuates edges would be expected to improve spatial vision and the detection of objects against a background. Luminance differences are certainly one way an edge can be defined (as shown in Figure 3). Of course, in the real world, things are rarely achromatic. 

**Figure 4:**
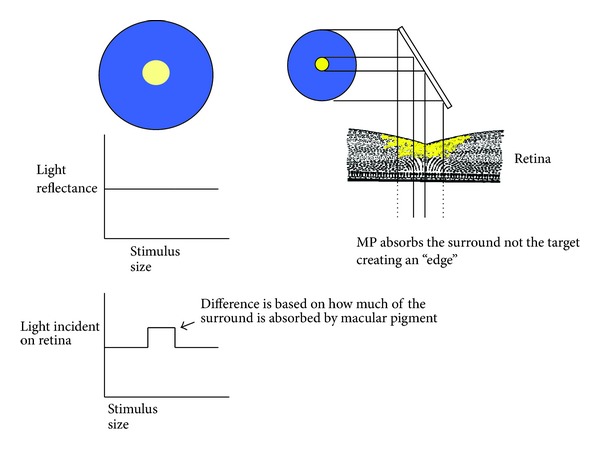
This example shows a blue surround with a yellow stimulus of equal luminance. Note that what defines the edge is based only on the wavelength or color difference. The luminance difference itself, however, will be exaggerated by differential absorption by macular pigment.

Consequently, other differences, such as wavelength composition (color), are used to define edges [53]. This is the reason that colored filters can make objects appear more “crisp.” Yellow filters, for instance, will make a yellow target with a blue surround more visible by selectively reducing the surround relative to the center. This simple optical effect enhances the *contrast* between a mid- or long-wave target and a background with more short-wave energy. See Figure 4.

**Figure 5:**
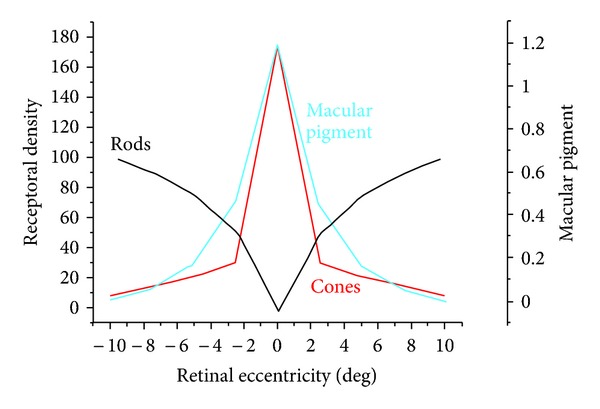
Data for the rod and cone densities were obtained from the original data by Osterberg, 1935. The MP distribution was obtained from Werner et al. 2000 who measured MP density with HFP using a 12-degree reference. Note that for this example, MP is screening a significant number of rods in the central macula (around 10 degrees in diameter).

Both Luria [102] and Wolfshonn et al. [103] have shown that the visibility of stimuli like these is improved when viewed through yellow lenses. We can also see such an effect when MP is measured directly; MP (also simply a yellow filter) selectively absorbs the background making the target more visible (Figure 5).

It seems fairly obvious that colored filters will enhance contrast whenever the wavelength difference between an object and its surround/background is enhanced by selective absorption by the filter [104–111]. When the luminance ratio between the target and a background is close, this process will be enhanced due to the phenomenon of brightness induction. Kvansakul et al. suggested another situation where contrast sensitivity might be enhanced, mesopic vision levels. In the LUXEA II trial, L, Z, and L and Z were supplemented and shown to improve contrast acuity [112].

Pérez et al. report a very similar effect of yellow filters on mesopic contrast acuity [113]. Such results make sense. Humans have duplex vision. We have cones in our central retina that mediate color vision, fine acuity, and so forth during the daytime when light levels are high. During this time, the photopigment of rods is effectively isomerized (bleached) and rods contribute little. At night (low light levels), however, the photopigment in rods regenerates and rods take over our visual function (we shift from photopic to scotopic vision, to use the visual science vernacular). Because there are so many rods (90 million or so) compared to cones (5 million or so), rods are more sensitive and therefore more useful at times when little light is available. (A probable reason for why vitamin E (transparent to visible light) is the primary antioxidant in the periphery, whereas carotenoids which filter visible light are in the center. To wit, we can afford to lose light in high-light circumstances.) There is a period, however, when both cones and rods contribute strongly to our visual experience. This period (usually around dusk and dawn in real world conditions) is known as mesopic vision. Kvansakul et al. [112] argue that, at such times, rods actually decrease contrast sensitivity. Rods do, in fact, have poorer contrast sensitivity and temporal resolution compared to cones. Kvansakul et al., suggest that high MP, by screening central rods, favors more cone-dominated mesopic vision which would confer superior contrast sensitivity. Empirical evidence has shown that yellow filters can improve motion sensitivity, convergence, and reading performance [114] presumably due to the fact that they influence the magnocellular system which receives its input from rods. Macular pigment does screen central rods as shown in Figure 5.

Kelly studied the phenomenon that yellow-tinted lenses appear to brighten the visual field [115]. She argued that this brightness enhancing effect of yellow lenses was due, in part, to the contribution of rod signals to the chromatic pathways. Based on its physical location, and the confluence of data from different sources, it can be concluded that yellow filters (like MP) can influence visual tasks that are mediated, in part, by rods. What we can also say is that it is clear that a tinted contact lens will increase contrast sensitivity when there is a wavelength difference between a central stimulus and its surround and this wavelength difference favors absorption by the filter (i.e., the contact absorbs one side of a chromatic edge more than the other). This is a strong visual effect but one could question whether it is ecologically valid. Hannsen and Gegenfurtner in an analysis of edges in the natural environment, noted that chromatic filters were as common as isoluminant (nonchromatic) edges [53]. 

How valid are the stimuli used in chromatic contrast studies? After all, how often does one view a mid- or long-wave (e.g., green, yellow, or red) target on a blue background? The answer, ironically, to this question is quite often. Understanding why this is so requires some discussion of atmospheric optics (next section). For a full discussion see Wooten and Hammond [26].

### 5.7. Visual Range

Luria conducted a very straightforward experiment and found a very predictable result [102]; the threshold for a yellow increment target on a blue background is reduced when viewed through a short-wave (yellow) filter (Wolffsohn et al. confirmed this effect using contrast measures [103]). Such an effect is obvious, that is, the blue background is selectively reduced by the yellow filters making the increment or contrast with the less absorbed target greater. At first, this effect appears trivial in that it seems that the stimulus is highly contrived and does not apply to very many examples of everyday vision. In fact, however, such a simple stimulus arrangement is a wonderful model for the optics of seeing objects in the distance. Wooten and Hammond performed an ecological analysis of stimuli in the environment [26] and argued that many objects viewed outdoors contain large amounts of mid- and long-wave light and are viewed on backgrounds that are short-wave dominant. The earth's atmosphere through which we view objects almost always contains small suspended particles from both natural and man-made sources. This *haze aerosol*, as it is called, scatters SW light more than other wavelengths and results in a bluish veiling luminance. *Blue haze*, as it is sometimes called, is a major factor that degrades *visibility*, that is, how well and how far we can see targets in the outdoors. Yellow filters may improve vision through the atmosphere by preferentially absorbing the SW energy produced by blue haze and, thereby, increasing both the contrast within targets and the contrast of targets with respect to their backgrounds.

Light scatter in the atmosphere is wavelength dependent, being strongest at short wavelengths (*λ* ^−4^, Rayleigh scatter). A similar effect occurs with haze. It is easily observable that distant objects, such as the features of mountain sides, and so forth, have a distinctively bluish appearance (e.g., purple mountains majesty). Hydrocarbon particles released by vegetation (such as terpenes) react with ozone creating “blue haze” that limits vision in the distance. The peak energy of blue haze and sky light is both 460 nm (the peak absorption of MP). 

 A somewhat opposite effect occurs for objects that are in our sight of line. Short-wave light is scattered out of the optical path and the wavelength composition of the target is shifted towards the longer wavelengths. 

 The net effect is that we are often viewing targets that are mid or long wave (not absorbed by a yellow filter like MP) with surrounds that are bluish (absorbed by yellow filters like macular pigment). Since, under such circumstances, macular pigment would reduce the background relative to the surround, the contrast or difference between the two would be accentuated. The more filtering, the more contrast would be enhanced. 

 Wooten and Hammond [26] mathematically modeled these effects and argued that MP, as a simple yellow filter, would improve vision in the atmosphere by about 30% (i.e., one could see about 30% farther distance) when comparing subjects with low and high MP. This estimate was similar to empirical data recently published by Hammond et al., 2012 [27]. These authors measured contrast sensitivity functions while imposing “blue haze” and while simulating changes in MP density using an artificial variable filter. 

### 5.8. Application to Sports Vision

Obviously, a consideration of vision outdoors is of particularly relevance to athletes such as baseball players. Most athletes perform at the highest level of their sensor and motor thresholds [116]. As such, a small improvement (as might be achieved by using appropriately colored goggles, by increasing MP density, etc.) can translate to large gains. Many types of athletic performance involve visual performance outdoors and might be expected to be improved by colored contact lenses. One example is baseball. Baseball players are constantly exposed to many situations where optimal visual capabilities are required. Baseball players, like many athletes who burn lots of calories due to excessive exercise, may have relatively poor diets, which typically do not include enough fruits and vegetables [117], and likely low macular pigment levels. Such players might therefore garner large improvements in performance by the relatively simple means of wearing tinted lenses or increasing MP density through focused changes in diet or supplementation.

## 6. Additional (Possible) Biological Effects of Colored Filters 

### 6.1. Influencing the Relative Activity of Visual Pathways

 It has long been recognized that certain disorders (visual dyslexia, schizophrenia, strabismic amblyopia, autism, etc.) can be characterized by an imbalance of activity in the various visual pathways (the magno-and- parvocellular systems have been most studied) [118–121]. It is for this reason, that colored filters, overlays, colored backgrounds, and so forth have long been suggested as curative for conditions like dyslexia [122]. In general, studies of the efficacy of filtering techniques have been largely mixed (more heavily weighted towards the not effective side, [122]). Many of our earlier criticisms can be leveled at this literature, largely conducted by clinicians (non-visual scientists): poor specification of the stimuli and filters, no consideration of internal filters like MP and the lens, and so forth. There are also large individual differences in the patient populations which means that one could expect the filters to influence different individuals differently. (A general clinical rule is that no disease is really a single homogeneuous entity. For example, an individual could have macular degeneration that was due variously to smoking or light exposure or genetics. In each case, they manifest somewhat similarly but have completely different etiologies. Even the manifestation of most diseases are so different that most are characterized statistically with a minimum set of criteria that each individual's disease is more or less consistent with. As a result, therapies for a given individual work differently; e.g., colored filters might work for some dyslexics but not others.) There are a few observations that are worth making. One is that it is clear that the nature of the light source/filtering will dramatically influence the relative activity of the parvo/magno pathways. It is also clear that this is sometimes imbalanced for some individuals. What is not clear is how to precisely align the specific characteristics of a filter with a given individuals imbalance. 

In any event, it probably should be expected that colored contacts will have some effects on visual processing that will benefit some, be negative for others, and likely neutral for yet others. Of course what would be optimal is to assess the specific imbalance that is present in a given condition and then to fashion chromatic filters that would correct this balance (analogous to testing refractive state and proscribing lenses). The technology to do this already exists, it simply needs to be made more facile.

### 6.2. Other Clinical Effects

Loss of visual function is both a prognostic and the worst outcome of visual disease. This is particularly true for conditions that affect the crystalline lens and retina (like age-related cataracts, ARC, and macular degeneration, AMD). Since treating the underlying disease is often so difficult (especially for conditions like macular degeneration), the approach is often palliative (e.g., correcting refractive errors or magnification). The use of prescription filters, however, is becoming increasingly common. These filters are largely aimed at reducing glare by absorbing short-wave light [123]. This is needed since disability due to glare is an exceptional problem for patients with even very early signs of cataract (due to increases in media scattering) and AMD. For example, Sandberg and Gaudio showed that when subjects with maculopathy are exposed to bright bleaching lights, visual recovery is significantly slowed despite having normal visual acuity [124]. Patients with early or more severe stages of AMD, for instance, tend to recover from a photostressor six to sixteen-times more slowly, respectively, than age-matched controls [125, 126]. 

It is not simply ocular disease [127] that manifests with visual symptoms. Numerous neurological diseases have distinctly visual symptoms. For example, greater than 1.6 million American warfighters have deployed to Iraq and Afghanistan in the last decade. Recent studies have estimated that about 20% of those returning from combat have sustained mild TBI, and that most of these cases go untreated [128–130]. The prevalence of mild TBI appears equally great in many sports that involve concussive incidents (like football). Like the more severe forms of TBI (a different clinical entity), the effects of mild TBI are very long lasting. Although diagnostic criteria tend to be inadequate, the most significant appear to be subtle visual effects. For example, a recent publication by CRC press (2011) was entitled “Vision rehabilitation: multidisciplinary care of the patient following brain injury” by Penelope Sutter and Lisa Harvey. This volume was dedicated to characterizing known visual deficits in patients with varying grades of post-concussive injury. Noted prominently in this volume was the following visual deficits: increased sensitivity to bright light (glare discomfort), increased visual disability due to glare, impaired temporal vision/motion processing, slowed photostress recovery. These are all visual disabilities that chromatic filters might be expected to improve.

### 6.3. Protection from Actinic Damage 

 There is an important additional function that chromatic filters likely perform. They protect the more vulnerable tissues in the posterior pole of the eye from damage due to actinic light [100]. There is little doubt that both the chromophores within the lens and macular pigment in the human eye protect from energetic short-wave light.

#### 6.3.1. Light and Oxygen

Early in the evolution of our atmosphere (The Precambrian period), there was no oxygen and all life was represented by photosynthetic anaerobes. Geological evidence (i.e., rust in rocks) suggests that blue-green algae started producing oxygen as a means of destroying other plant-like organisms that were competing in the harsh environment of a new world. These plants, in turn, developed antioxidants to protect themselves from this, essentially, toxic gas. Animals, in general, also evolving in an environment with about 21% oxygen, took advantage of this age-old defense of plants, antioxidants. Of course oxygen in the atmosphere is normally inert. It takes an energy source to convert this inert oxygen into the more reactive forms that are capable of damaging biological tissue.(Reactive oxygen is often described as a free radical (an atom/molecule with an unpaired electron in its outermost orbit) but, strictly speaking, reactive oxygen is not a free radical. Oxygen in its ground-state or triplet form (^3^O_2_) is a diradical; meaning that it possesses two unpaired electrons spinning in coordinated parallel orbits. In this form, oxygen is relatively stable. If triplet oxygen, however, absorbs enough energy to reverse the spin of one of its unpaired electron orbits (e.g., by absorbing short-wave light), it can convert to a more reactive singlet form (^1^O_2_). The singlet form of oxygen stays reactive for a relatively long time period since conversion back to the triplet form is spin forbidden.

Receptoral outer segements contain high quantities of polyunsaturated fatty acids (PUFA). If reactive oxygen species are not quenched, they can peroxidize membrane lipids. For instance, reactive oxygen species can abstract hydrogen atoms from PUFA-rich receptoral membranes. The withdrawal of hydrogen will convert the PUFA into an organic radical that may then react in a similar manner with adjacent PUFA molecules.) Light is an energy source that is often focused directly on retinal tissue making it (in the presence of a photosensitizer, like photopigment) one of the more significant stressors.

 Visible light, of course, is just a very small portion of the electromagnetic spectrum. Humans perceive the portion from about 400 to 700 nm (a billionth of a meter). Such light is not equally capable, however, of damaging retinal tissue. This is because the energy of light is inversely proportional to its wavelength; longer wavelengths are less energetic than shorter wavelengths. For example, heating metal will originally glow red, then slowly as the electrons in the metal become more active, the metal will glow white hot (it will emit a mix of all wavelengths). As applied to the eye, light from about 500–700 nm can damage the retina, but only through thermal mechanisms (although it can increase the potential that shorter wavelengths will be damaging because it raised the overall energy state). An enormous amount of light would be necessary to heat the retina up enough to cause damage.

Empirical evaluations (mostly using animal models) have shown that light from about 400–500 nm is the most damaging to retinal tissue because (1) it reaches the retina and is not significantly absorbed by anterior structures; (2) it still retains enough energy to initiate photochemical damage (e.g., convert inert oxygen into reactive forms); (3) it fits the action spectrum of retinal photosensitizers. As an example, of the latter, Margrain et al. argued [100] that this fact makes short-wave light even more damaging to the elderly who contain higher levels of some photosensitizers (although less of others, like less photopigment). 

Knowing the action spectrum for light damage to the retina is quite important, of course. Many professionals use light that could potentially be damaging. For example, ophthalmologists use blue-green argon lasers for photocoagulation and dentists use blue lasers to cure dental compound. There is also some concern that normal and accidental light exposures could damage ocular structures. As an example of the latter, many have expressed concern that accidental exposure to laser pointers could damage the retina. As the foregoing should demonstrate, however, this is unlikely. Laser pointers are almost always classified as class 2 or 3a lasers (lasers are simply light of a very narrow or single wavelength) which have a power output of less than 5 mW. As noted, laser pointers are typical red which is far outside the action spectrum for photic injury. Normal exposure would be less than a second (warning labels on lasers usually apply to holding them directly to the eye and staring at them for at least ten seconds) and would be terminated by normal aversive responses (blinking or looking away). 

**Figure 6:**
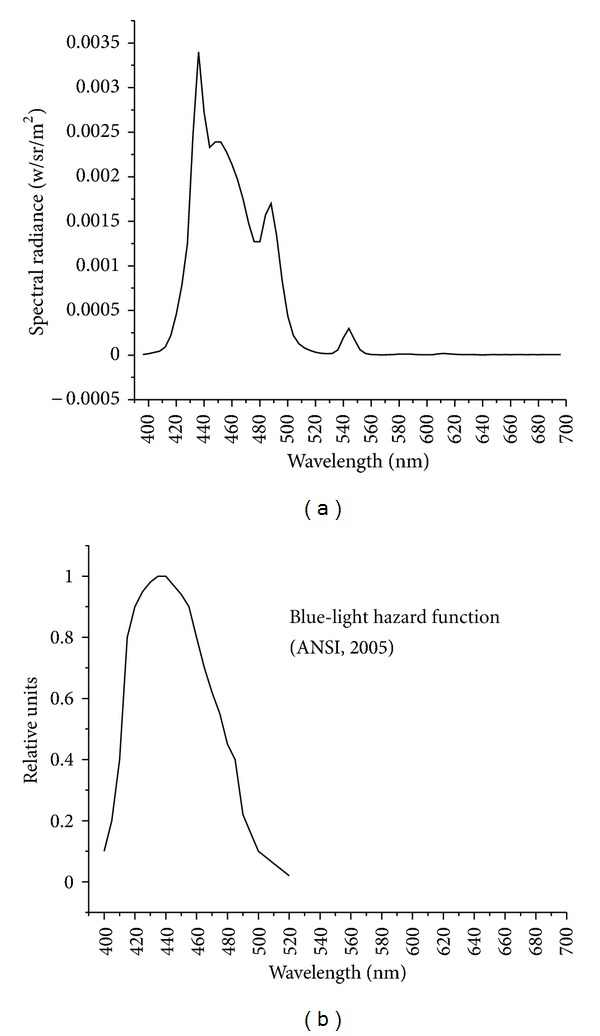
Light emitted by a computer monitor set to a blue background (a) compared to the blue-light hazard function published by ANSI.

A different situation would be the light of computer or television monitors. Individuals do stare at monitors for periods that can last as long as an entire day. Moreover, monitors certainly can emit light that fits the general photic hazard profile. The following graphs display the light hazard function published by ANSI next to the spectral emission characteristics of my computer monitor (a standard LCD display) set to a solid blue background. See Figure 6. As shown in the left side of the figure (measurements were taken while the monitor was set to a blue background color), computer monitors can easily emit light specific to the most damaging region of the visible spectrum. At issue, however, is the fact that the standard radiance level of most monitors is very low. Most monitors emit light at around 10 cd/m^2^. Ambient illumination is usually about 10–20 cd/m^2^. In other words, you would get about as much exposure staring at a blue wall, which reflects short-wave light into your eye, as you would by staring at a blue (light-emitting) monitor. Indeed, individuals with professions outdoors would get far more damaging light exposure than an office worker staring at their monitor all day (which is rarely optimized to fit the photic damage spectrum). 

## 7. Conclusion

 Humans possess a yellow-filtering pigment in the inner layer of the retina that deposits in and around the fovea and gives that area its clinical designation as the macula lutea. As we age, the crystallin proteins within the lens oxidize creating yet another intra-ocular yellow filter. The presence of yellow intra-ocular filters is likely one of the older adaptations of our eye and one that we share with many other diurnal species including fish, squirrels, tree shrews, snakes, geckos, lampreys, and so forth. These filters appear to have many positive functions in photopic vision. To quote Walls, [[131], pages 95-96].

*“Glare and dazzle are minimized by a yellow filter. Similarly, the unfocusable short-wave light scattered in the atmosphere, and responsible for the bluish cast of distant mountains and for the blue of the sky, is cut out by a yellow filter which, as every photographer knows, creates a sharper image. Still another effect is the enhancement of contrast.” *

By absorbing one side of a chromatic border more than the other, the difference (or contrast) is enhanced. This has wider application than may at first be appreciated. Often adjoining objects in nature appear similar in color but actually a spectral analysis would show that the two are quite different. An effect of colored filters on chromatic contrast is, however, a mixed effect; it is as likely to reduce contrast as often as it is to enhance it. This point was also addressed by Walls and was, again, used as an argument for why yellow was the pigment most often found in diurnal species.

*“By cutting out the different amounts of blue in different but alike-looking green mixtures, the greens are made to look unlike; and almost any other contrasts can be sacrificed by the animal if only those between greens, so numerous in nature, can be enhanced.” *

The macular pigment of the human eye is a major feature of the fovea. The slow yellowing of the crystalline lens happens to all individuals as they age. It is likely that our intraocular filters do what they do in most species, protect ocular tissues and improve vision under ecological conditions. Efforts to create tinted extrinsic (spectacle lenses, performance goggles) or intrinsic (IOLs) filters should base their design on the designs already provided by nature, the lens and macular pigmentation.

## Abbreviations

MP: Macular pigment
L: Lutein
Z: Zeaxanthin
OD: Optical density
B: Blue
G: Green
R: Red.
